# Utilization of Microfluidic Droplet-Based Methods in Diagnosis and Treatment Methods of Hepatocellular Carcinoma: A Review

**DOI:** 10.3390/genes15101242

**Published:** 2024-09-25

**Authors:** Akvilė Zajanckauskaite, Miah Lingelbach, Dovilė Juozapaitė, Algirdas Utkus, Greta Rukšnaitytė, Goda Jonuškienė, Aistė Gulla

**Affiliations:** 1 Department of Human and Medical Genetics, Faculty of Medicine, Vilnius University, 01513 Vilnius, Lithuania; 2 School of Osteopathic Medicine, A.T. Still University, Mesa, AZ 85206, USA; miah.lingelbach@atsu.edu; 3 Vilnius Santaros Klinikos Biobank, Vilnius University Hospital Santaros Klinikos, 08661 Vilnius, Lithuania; 4 Droplet Genomics, 10223 Vilnius, Lithuania; 5 Clinic of Hematology and Oncology, Institute of Clinical Medicine, Faculty of Medicine, 01513 Vilnius, Lithuania; 6 Institute of Clinical Medicine, Faculty of Medicine, Vilnius University, 01513 Vilnius, Lithuania; 7 Department of Surgery, George Washington University, Washington, DC 20052, USA

**Keywords:** single-cell analysis, hepatocellular carcinoma, heterogeneity cancer liver sequencing

## Abstract

Hepatocellular carcinoma (HCC) is one of the most common cancers worldwide and is associated with high morbidity and mortality. One of the main challenges in the management of HCC is late clinical presentation and thus diagnosis of the disease, which results in poor survival. The pathogenesis of HCC is complex and involves chronic liver injury and genetic alterations. Diagnosis of HCC can be made either by biopsy or imaging; however, conventional tissue-based biopsy methods and serological biomarkers such as AFP have limited clinical applications. While hepatocellular carcinoma is associated with a range of molecular alterations, including the activation of oncogenic signaling pathways, such as Wnt-TGFβ, PI3K-AKT-mTOR, RAS-MAPK, MET, IGF, and Wnt-β-catenin and *TP53* and TERT promoter mutations, microfluidic applications have been limited. Early diagnosis is crucial for advancing treatments that would address the heterogeneity of HCC. In this context, microfluidic droplet-based methods are crucial, as they enable comprehensive analysis of the genome and transcriptome of individual cells. Single-cell RNA sequencing (scRNA-seq) allows the examination of individual cell transcriptomes, identifying their heterogeneity and cellular evolutionary relationships. Other microfluidic methods, such as Drop-seq, InDrop, and ATAC-seq, are also employed for single-cell analysis. Here, we examine and compare these microfluidic droplet-based methods, exploring their advantages and limitations in liver cancer research. These technologies provide new opportunities to understand liver cancer biology, diagnosis, treatment, and prognosis, contributing to scientific efforts in combating this challenging disease.

## 1. Introduction

**Figure 1:**
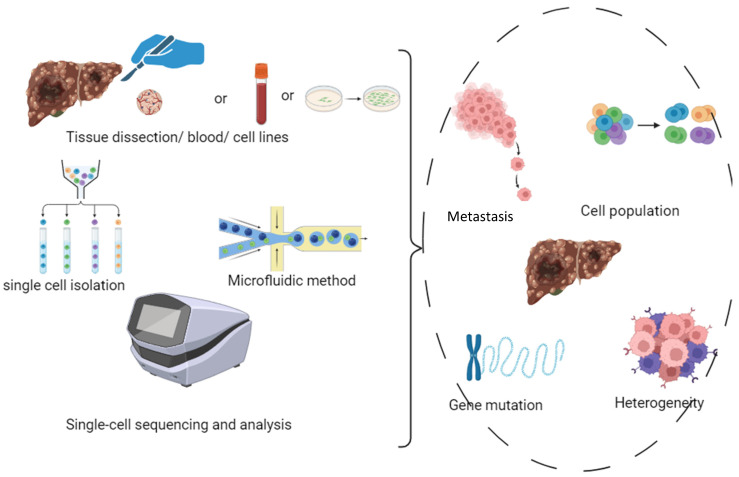
Explanation of intracellular analysis that can be observed via various single-cell analysis methods. Image designed by app.biorender.com.

Liver cancer is one of the most common forms of cancer [1]. Recent studies show that liver cancer is one of the four leading causes of cancer-related deaths [2]. Liver cancer is divided into two types: primary and secondary. Primary liver cancer is a malignant tumor that arises in the liver, such as hepatocellular carcinoma, cholangiocarcinoma, or angiosarcoma. There is also a secondary form of cancer that starts elsewhere in the body but spreads to the liver [3]. Primary cancers account for about 4.7% of all cancers. The most common patients are male, with the highest incidence in the 45–60 age group [4]. The liver is the main organ that supports metabolism, digestion, immunity, and detoxification of the body. Although the liver has an essential capacity for regeneration, these functions can be affected by continuous exposure to various chemicals. Since the early stage of liver cancer is asymptomatic and symptoms occur mainly in the later stages, it is essential to detect and diagnose the disease in time and to take all possible measures to stop the disease [5]. Treating liver cancer is very challenging, as it is often diagnosed at an advanced stage and is potentially unresponsive to drug treatment [6]. Hepatotoxicity and liver damage are the main drug-induced consequences of late-stage cancer. Thus, malignant tumor progression and metastasis are causes of high mortality [7]. Studies have shown that genetic and genomic variation in tumor tissue can lead to cells with different genetic and phenotypic characteristics, resulting in highly heterogeneous tumor tissue [8]. Mutations in liver cancer cells can lead to abnormal cell growth, leading to tumor development [9,10]. Liver cancer can be associated with a number of genetic mutations, such as a mutation in the *TP53* gene, which regulates cell growth, apoptosis, and DNA repair. A mutation in the *CTNNB1* gene is important for a signaling pathway that regulates biological processes in the body. Other mutations that affect the development of cancer are also involved [11]. High heterogeneity may be associated with the mechanism of tumorigenesis and metastasis. For this reason, a more precise analysis of tumor cells is performed. Traditional sequencing methods are not suited to analyzing a portion of cells and detecting heterogeneity and therefore have significant drawbacks in the analysis of tumor cells. Single-cell sequencing technologies can perfectly compensate for the shortcomings of traditional sequencing methods. Single-cell sequencing technologies allow the sequencing of the genome or transcript of a single cell in order to obtain genomic, transcriptomic, or other multifunctional information that would reveal differences in the cell population and the evolutionary relationships among cells [3,12]. As research continues to deepen, the potential of single-cell sequencing techniques continues to grow and evolve [13]. The advent of single-cell sequencing has had a major impact on the field of cancer research as it has improved our understanding of tumor heterogeneity, the tumor microenvironment, metastasis, and resistance to treatment [14]. Therefore, single-cell sequencing studies are of great value in cancer research, and their results are contributing to the development of the field of personalized medicine. Liver cancer is highly heterogeneous at both the molecular and histological levels, and high-throughput sequencing and gene expression profiling can identify genetic alterations and specific gene mutations. These studies can help us to understand the biology of liver cancer, genetic mutations, and the application of this knowledge in clinical practice [15]. As the mortality rate of liver cancer continues to increase, these studies, when applied in medical practice, can change the treatment of the disease [16]. Figure 1 exemplifies this in a picture for further clarification.

## 2. Liver Cancers and Their Gene Mutations

The liver is the main organ that regulates the elimination of toxins; balances the absorption of glucose, lipids, and amino acids; regulates the metabolism of the whole body; and maintains metabolic homeostasis. Tumor initiation and progression are influenced by disturbed metabolism, [17] drug metabolism, digestion, or detoxification [18,19]. Delayed diagnosis is a major cause of high mortality [20]. According to the latest Globocan Global Cancer Statistics Report, in 2020, 905,700 people worldwide were diagnosed with liver cancer and 830, 200 people died from liver cancer. Based on annual projections, the World Health Organisation estimates that 1,276,679 patients will die from liver cancer in 2040 [4,21]. Hepatocellular carcinoma (HCC) is the most common type of primary liver cancer and accounts for about 80–90% of all primary liver cancers. The most common causes of HCC are viral hepatitis (hepatitis B or C virus), alcohol, smoking, and diabetes mellitus [22,23]. The second most common liver carcinoma after HCC is intrahepatic cholangiocarcinoma (ICC), which accounts for about 15% of primary liver cancers, with an incidence of 2 per 100,000 population worldwide each year [21]. The most common risk factors for ICC are biliary tract disease, gallstones, viral hepatitis (hepatitis B or C virus), metabolic syndrome, cirrhosis, and tobacco or alcohol use [24]. Liver cancer is caused by genetic mutations that are linked to epidemiological conditions [25,26]. This lethal malignancy is characterized by heterogeneity, which is considered one of the main reasons for the development of drug resistance and the failure of clinical trials [27]. Single-cell sequencing technologies allow the analysis of each cell in a tumor tissue sample, providing a complete understanding of the genetic heterogeneity of the tumor, which helps to identify and evaluate rare cell populations by analyzing the gene expression pattern differences between individual cells in a single biopsy tissue, which are not usually identifiable by the pooled cellular gene expression pattern (traditional sequencing technologies) [8,28]. The different morphological phenotypes of HCC are associated with different genetic changes that promote tumor progression. The development of high-carrier sequencing technologies has allowed a comprehensive genetic profile of primary liver cancer to be developed [29]. These studies have shown that HCC patients have hundreds of somatic DNA alterations, including chromosomal aberrations or mutations [30,31].

To date, the most common alterations identified in HCC are mutations in the TERT promoter, *CTNNB1,* and *TP53* [31]. *TP53* mutations account for about 30% of cases of HCC, while mutations in genes involved in WNT signaling (*CTNNB1* and *AXIN1*) and chromatin remodeling (*ARID1A*) account for 27–40% [32,33,34,35]. In ICC, the most common genetic mutations are *TP53*, *KRAS*, *ARID1A*, *BAP1*, *IDH1*, *IDH2*, *PIK3CA*, *SMARCB1*, *EPHA2*, *SMAD4*, *GNAS* and *PBRM1*, and *FGFR* [7]. *KRAS* and *TP53* are among the most frequently mutated genes in ICC [35,36].

## 3. Methods of Microfluidic Droplet-Based Cell Analysis

### 3.1. Overview of the Droplet Microfluidic Process

One of the fluid manipulation techniques used in microfluidic technology is droplet microfluidics. This technology is based on the generation of droplets of the micrometer order of magnitude in diameter and their manipulation in channels of the micrometer order of magnitude [37]. The formation of droplets in these dimensions is induced by the viscous and surface tension forces of the fluid. These physical forces result in the mixing of two immiscible liquid phases in the microchannels to form an emulsion—a two-phase dispersive system. One of the simplest emulsions is oil droplets in water [20]. The main advantage of this technology is the ultra-high-throughput compartmentalization of reactions (droplets are generated at ~1000 droplets/second) and analysis (~100–1000 droplets/second). Various microfluidic chips are applicable in this field, such as droplet generation, droplet sorting, droplet merging, and reagent addition, depending on the experiment being performed. Chip-generated droplets have several advantages: firstly, produced droplets are monodisperse and identical, allowing a large number of reactions to be analyzed [38]; secondly, droplets are biocompatible, i.e., not toxic to the cells or other biological objects placed in them, which ensures the analysis of uninfluenced biological objects. Thirdly, droplets have a high volume-to-area ratio, which results in a much faster material and heat exchange, making reactions more efficient [39]. Additionally, smaller quantities of reactants are used as the droplet volume is small (micro-, nano-, -pico- scales). Finally, it is possible to encapsulate a single cell/molecule in the droplets, which allows single-cell/molecule analysis [37]. Droplet microfluidic technology can be used to analyze the transcriptomes of single cells. This is made possible by encapsulating cells in nanoliter droplets together with barcoding DNA primers (immobilized on hydrogel beads) and enzymatic reaction components for RNA amplification. The encapsulation process shall ensure that one cell and one hydrogel particle are statistically present per droplet [40]. When a hydrogel particle and a cell are loaded into the same droplet, the latter is lysed and released mRNA molecules are tagged with a unique barcode readable by sequencing [41,42]. Droplet microfluidics technology is commonly used in the biomedical sciences, including single-cell genetics, oncology, immunology, and microbiology. Due to its ability to perform high-sensitivity analysis, this technique is highly valued in studies related to cancer cell heterogeneity, the identification of rare cells, as well as in the analysis of infectious pathogens. Single-cell transcriptome analysis can reveal differences in gene expression in a population of cells, which is important for understanding the pathogenesis of disease, for personalized medicine, and for the development of new treatments for disease [13]. This is summarized in Table 1.

### 3.2. ATAC—Assay for Transposase-Accessible Chromatin Sequencing Method

ATAC-seq is a technology which stands for Assay for Transposase-Accessible Chromatin using sequencing [46,47]. This technology is used in molecular biology to assess genome-wide chromatin accessibility [48]. ATAC-seq works by using a transposase enzyme to cut the DNA at accessible regions. The transposase enzyme is a protein that can cut DNA at random positions and at the same time tag it with DNA sequences of choice. However, tightly packed DNA is less prone to transposase binding [48,49]. The DNA fragments that are produced by the transposase enzyme are then sequenced. The sequencing data can be used to identify the regions of the genome that are accessible to the transposase enzyme [50]. These regions are considered to be open chromatin, and they are the regions where transcription factors can bind and regulate gene expression [51]. The accessibility of chromatin refers to how tightly the DNA is wrapped around the proteins [52] and is regulated by a variety of factors, including DNA methylation, histone modifications, and chromatin remodeling. DNA methylation is a chemical modification of DNA that can silence genes. Histone modifications are chemical changes to the proteins that package DNA [53]. Chromatin remodeling is the process of changing the structure of chromatin. Changes in chromatin accessibility can affect gene expression. For instance, if a region of chromatin becomes more accessible, it is more relevant that transcription factors will bind to that region and regulate gene expression [54]. Analyzing an open chromatin would help us to understand how genes are regulated and how diseases are developed [55,56].

ATAC-seq could be a useful tool for liver cancer analysis. This technology has been applied in a number of studies to identify genes and pathways that are dysregulated in liver cancer cells. One of the studies showed that scientists could compare the chromatin accessibility of liver cells by using the ATAC-seq method. The researchers found that several genes were differentially accessible in the cancer cells, including genes involved in cell metabolism, proliferation, and apoptosis [57]. Changes in chromatin accessibility were associated with specific DNA methylation patterns [58]. This indicates that DNA methylation may play a role in regulating the expression of genes in liver cells [59]. Researchers applied the ATAC-seq method for liver cancer prognosis. This method let them identify 15 signature genes (*PRDX6*, *GCLM*, *HTATIP2*, *SEMA3F*, *UCK2*, *NOL10*, *KIF18A*, *RAP2A*, *BOD1*, *GDI2*, *ZIC2*, *GTF3C6 SLC1A5*, *ERI3,* and *SAC3D1)* that are overexpressed in hepatocellular carcinoma [54,57]. These genes are highly expressed in cancerous tissues and are associated with poor patient prognosis [60]. Also, genes are correlated with tumor purity and immune cells infiltration levels, suggesting that these genes might play a role in tumor progression by regulating the tumor microenvironment [61]. Another study used ATAC-seq to identify enhancers that were active in HCC cells. Enhancers are DNA sequences that regulate the expression of genes, and they are often found to be mutated in cancer cells [49]. The study found that a number of enhancers that were active in HCC cells were also active in other types of cancer [62], suggesting that they may play a role in cancer progression. The results of ATAC-seq can be used to identify genes and pathways that are dysregulated in HCC. This information can be used to develop new diagnostic and therapeutic strategies for HCC [59,63].

Overall, ATAC-seq is a powerful technique with a wide range of applications [64,65]. ATAC-seq can be used to identify the regulatory elements that are important for the new progression and development of HCC cells. This information could be used for studying the epigenetic landscape of HCC cells and developing new therapeutic strategies and diagnostics [60,65].

### 3.3. InDrop

InDrop is a droplet microfluidics method for single-cell analysis, allowing the identification and analysis of thousands of single cells simultaneously. The method is widely used in research, including in the field of diagnostics, cancer diagnosis, and treatment prognosis [39]. The basic principle of the technology is that a mixture of cells is encapsulated in microfluidic droplets together with oligonucleotide primers, RT, and lysis reagents. The mRNA released from the lysed cells remains in the same droplet and is labeled with the oligonucleotide primers during the RT reaction. After barcoding, all cellular material is pooled and the cDNA library is processed for next-generation sequencing. The InDrop method is unique in that it traps individual cells in a single drop of microfluidics. This allows for the efficient analysis of the cell genome and transcriptome (gene expression) and the identification of single-cell heterogeneity [66]. This is important, especially given that liver cancer can be of different subtypes with different gene expression profiles [40].

For the diagnosis of liver cancer, the InDrop method allows the separation of healthy liver cells from the cancer cell population, the identification of specific cancer markers and subtypes, and the assessment of cellular heterogeneity. This provides valuable information on cancer progression and the possibility of personalized treatment. The InDrop method for liver cancer diagnosis first involves isolating single cells from the affected liver tissue. These cells are then captured in a microfluidics droplet where each cell is assigned a unique sequence barcode. In the next steps, the cells are lysed and the resulting mRNA is analyzed to determine their gene expression levels [67]. The InDrop approach allows the identification of specific markers of liver cancer, including genes that may be involved in cancer growth, invasion, and metastasis [68]. It also allows the identification of clonal subtypes and heterogeneities of liver cancer, which provides information on cellular diversity and helps to determine prognosis [66]. One of the differences in the InDrop method is that it allows you to load almost 100% of one bead in one drop. This ensures that individual cells that randomly enter the droplet are exposed to a single unique DNA barcode, which is particularly important when capturing rare cells [28,66]. One of the advantages of this method is that it is possible to process thousands or tens of thousands of cells. The InDrop method can capture cells of any size. This technology allows the detection of large numbers of cellular scRNA-seq, which allows the identification of very rare cell types from heterogeneous populations [69]. It is also possible to capture thousands of cells in less than an hour, which is a short time, and 1000 cells in a few minutes. The InDrop method is also suitable for the analysis of very small tissue samples as a high percentage of cells are captured [70].

Also, one of the biggest advantages of this method over other single-cell sequencing methods is that it is less expensive. Despite the great advantages of this method, there are some disadvantages. InDrop sensitivity measurements show that this method is three times less sensitive compared to lower-throughput methods. Thus, if the differences in the cells to be tested are important and subpopulations of cells of interest are not rare, lower-throughput methods should be the first choice, but further refinement of this method will only increase its sensitivity [40]. In conclusion, InDrop microfluidics is a powerful technology for single-cell analysis, including liver cancer diagnostics. Its application in this field helps us to understand the biology, diagnosis, treatment, and prognosis of liver cancer, promoting progress in the fight against this serious disease [71].

### 3.4. Drop-Seq

**Figure 2:**
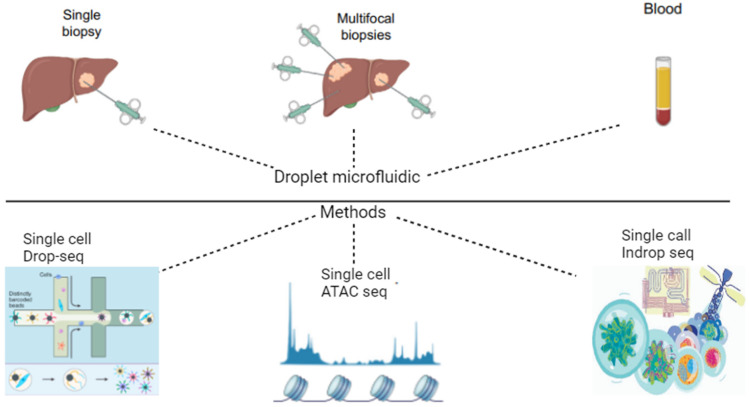
Ways samples of droplet microfluidic samples can be obtained and methods that could be used for analysis. Image designed by app.biorender.com.

Drop-seq is a single-cell RNA sequencing (scRNA-seq) technology that allows for the analysis of thousands of individual cells in parallel. It was developed by Macosko and his team in 2015 [72,73]. This technology works by encapsulating single cells and barcoded microparticles in nanoliter-sized droplets. The cells and beads are diluted such that only a few droplets will contain a bead, a cell, or both, like the InDrop method. This enables a very low doublet rate (the percentage of droplets that contain two cells) but results in lower cell capture efficiency (the percentage of cells that are successfully encapsulated in droplets). Once the cells are encapsulated, they should be immediately lysed, which causes the release of polyadenylated mRNA transcripts. The droplets are then broken, and the mRNA is reverse transcribed, forming covalent and stable STAMPs (single-cell transcriptomes attached to microparticles). Exonuclease treatment is then applied to remove bead primers that have not captured an mRNA molecule. cDNA amplification, library construction, and sequencing are then performed [74]. Drop-seq technology is similar to InDrop technology. Both methods use microfluidics to encapsulate single cells in droplets, but they differ in the way they label the cells. In Drop-seq, cells are labeled with barcoded microparticles, and InDrop uses barcoded hydrogels, capturing more cells than Drop-seq [75]. These methods are illustrated in Figure 2. Both technologies are powerful scRNA-seq tools that could provide more information about biological mechanisms, immune response, cancer, disease progression, cell differentiation [42,76]. Drop-seq is a suitable technology for liver cancer analysis, because it is possible to study the transcriptome of a single cell of liver cancer tissue. Drop-seq has been used to study the expression of genes involved in cell proliferation, metabolism, and apoptosis in liver cancer cells [26,77]. It has also been used to study the heterogeneity of liver cancer cells and to identify subtypes of liver cancer cells with different gene expression profiles. One of the studies shows that single-cell transcriptomics of hepatocellular carcinoma helps to identify novel therapeutic targets [44,78]. This article reveals that targeted therapeutic drugs, such as multi-kinase inhibitors, have limited benefits for patients with advanced HCC. Because of this, antibody-based cancer therapeutics are being developed as more precise and effective treatments for HCC. Drop-seq could be used for studying the developmental trajectory of HCC cells from healthy liver cells to precancerous cells to malignant tumor cells. Information about this can be used to identify new biomarkers for the early detection of HCC and develop new therapy pathways which would target the specific stages of HCC [28,70]. scRNA-seq is a promising tool for identifying new cancer biomarkers and therapeutic targets. By using this technology, it would be possible to generate the gene expression profiles of individual cells, which would be important because HCC tumors are very heterogeneous. Also, this technology is relatively inexpensive and easy to perform, and because of this, it would be easy to apply to the analysis of HCC [79].

## 4. Review of Literature

This review includes 100 scientific articles; the analysis of these articles focuses on the application of single-cell sequencing and droplet-based methods in hepatocellular carcinoma research. The analysis of these articles shows that in recent years, there has been a surge of interest in single-cell sequencing techniques for the study of cancer cells, cancer heterogeneity, and genomic and transcriptomic analysis, and thus for the development of personalized medicine. ATAC-seq is used to determine chromatin accessibility and the location of regulatory elements such as promoters and enhancers at the genome level [57,58,59]. In HCC research, this approach allows the identification of epigenetic changes that contribute to cancer development. InDrop and Drop-seq methods are used for single-cell transcriptome sequencing, allowing for the detailed analysis of cellular heterogeneity in HCC tissue [26,71,77]. The methods allow the separation of different cell subpopulations and the identification of their specific gene expression profiles. Table 2 summarizes the advantages and disadvantages of each method of single-cell analysis. The literature describes that HCC cell heterogeneity is higher than previously thought, which creates new opportunities for personalized therapy. While these single-cell research models offer great potential in HCC research, they also present some challenges in terms of data processing, interpretation, and integration into clinical practice. Further technological evolution and algorithm development are needed to efficiently handle and analyze huge amounts of data.

## 5. Conclusions

Applying single-cell technologies to liver cancer research would be the key to personalized medicine. Single-cell research models are increasingly being used to analyze various cancer forms, including HCC. The single-cell research models reviewed in this article like Drop-seq, InDrop, ATAC-seq, and more single-cell technology diagnostics provide insight into nucleic acid sequences within the tumor cell landscape, which can improve understanding of tumor resistance to treatment, leading to the development of ideal combination therapies [80,81]. Different cell types could potentially be recognized in HCC tumors and allow for new drug targets to be identified. Single-cell sequencing data can provide more than conventional genomic mutation data or gene expression data for predictive analysis. Improvements in existing single-cell sequencing technologies, the emergence of new techniques, and the integration of single-cell sequencing with other experimental protocols provide powerful tools to understand many of the remaining mysteries of cancer [81,82].

## Figures and Tables

**Table 1:** Literature summary of advantages and disadvantages of single-cell sequencing, 2021–2023.

Article	Advantages	Disadvantages
An Overview on Single-Cell Technology for Hepatocellular Carcinoma Diagnosis [43]	Provides a detailed understanding of heterogeneity in hepatocellular carcinoma tissues, the identification of novel biomarkers and rare cell types, and insight into the tumor microenvironment and cancer evolution	These are complex and costly technologies that require advanced technology to analyze the large amounts of data generated
Understanding tumor cell heterogeneity and its implication for immunotherapy in liver cancer using single-cell analysis [26]	Uncovers cellular diversity and complexity in tumors, providing insights into tumor evolution, clonal dynamics, and cellular interactions.	Challenges in developing cost-effective methodologies for data analysis and interpretation
	The technology allows the detection of cellular heterogeneity in tumors, the discovery of rare cell populations that may be missed in large-scale studies, and detailed insights into the molecular mechanisms underlying cancer progression and response to treatment	Complex data analysis, requiring advanced technological tools to process large datasets
Single-Cell Sequencing and Its Applications in Liver Cancer [28]	Uncovers tumor heterogeneity, identifies rare cell populations, and provides insights into tumor evolution and microenvironment	Technically challenging methods due to the need for accurate cell isolation and amplification techniques, and potential difficulties in data analysis due to the huge amount of data generated
Single-cell transcriptome sequencing reveals potential novel combination of biomarkers for antibody-based cancer therapeutics in hepatocellular carcinoma [44]	New biomarkers can be identified for antibody-based cancer therapies. It is possible to dissect tumor heterogeneity, to identify patterns of gene expression in different components, and to uncover gene signatures that can serve as biomarkers for the identification of dominant subpopulations during tumor evolution	
Single-cell analysis reveals the intra-tumor heterogeneity and identifies MLXIPL as a biomarker in the cellular trajectory of hepatocellular carcinoma [45]	Gene expression profiling of individual cells from tumor and paratumour tissues can be performed, revealing heterogeneity and potential therapeutic targets, e.g., MLXIPL	

**Table 2:** Summary of comparison of advantages and disadvantages of using ATAC-seq, InDrop, and Drop-seq in liver cancer research.

Technology	Advantages	Disadvantages
ATAC-seq	Efficient at assessing genome-wide chromatin accessibility - Identifies open chromatin regions for gene regulation study - Useful in identifying dysregulated genes and pathways in liver cancer - Can correlate chromatin accessibility with DNA methylation patterns - Helps in prognosis and development of new therapeutic strategies	- An expensive method - Difficult analysis; only 2–3% of genome is accessible
InDrop	- Allows analysis of thousands of single cells simultaneously - InDrop method enables efficient analysis of the cell genome and transcriptome - Identifies cancer markers and subtypes - Captures cellular heterogeneity - Suitable for analyzing small tissue samples - Less expensive compared to other single-cell sequencing methods	- Lower sensitivity compared to lower-throughput methods - May not capture all cell subpopulations
Drop-seq	- Analyzes thousands of individual cells in parallel - Low doublet rate - Useful in studying gene expression in liver cancer cells - Helps in identifying novel therapeutic targets - Relatively inexpensive and easy to perform	- Lower cell capture efficiency compared to InDrop - Limited by the need for immediate cell lysis after encapsulation

## Data Availability

All data are public and available to access for free.

## Abbreviations

HCC	Hepatocellular carcinoma
Wnt-TGFβ	Wnt-Transforming Growth Factor β
PI3K-AKT-mTOR	Phosphatidylinositol-3-Kinase—AKT (Protein Kinase B)—Mammalian Target of Rapamycin
RAS-MAPK	Rat Sarcoma—Mitogen-Activated Protein Kinase
MET	Mesenchymal–Epithelial Transition (or MET Proto-Oncogene)
IGF	Insulin-like Growth Factor
Wnt-β-catenin	Wnt-β-catenin
TP53	Tumor Protein p53
TERT	Telomerase Reverse Transcriptase
CTNNB1	Catenin β 1
KRAS	Kirsten Rat Sarcoma Viral Oncogene Homolog
ARID1A	AT-Rich Interaction Domain 1A
BAP1	BRCA1-Associated Protein 1
IDH1	Isocitrate Dehydrogenase 1
IDH2	Isocitrate Dehydrogenase 2
PIK3CA	Phosphatidylinositol-4,5-Bisphosphate 3-Kinase Catalytic Subunit α
SMARCB1	SWI/SNF-Related, Matrix-Associated, Actin-Dependent Regulator Of Chromatin, Subfamily B, Member 1
EPHA2	EPH Receptor A2
SMAD4	SMAD Family Member 4
GNAS	GNAS Complex Locus
PBRM1	Polybromo 1
ICC	Intrahepatic Cholangiocarcinoma
PRDX6	Peroxiredoxin 6
GCLM	Glutamate-Cysteine Ligase, Modifier Subunit
HTATIP2	HIV-1 Tat Interactive Protein 2, 30 kDa (also known as TIP30)
SEMA3F	Semaphorin 3F
UCK2	Uridine-Cytidine Kinase 2
NOL10	Nucleolar Protein 10
KIF18A	Kinesin Family Member 18A
RAP2A	RAP2A, Member of RAS Oncogene Family
BOD1	Biorientation of Chromosomes in Cell Division 1
GDI2	GDP Dissociation Inhibitor 2
ZIC2	Zic Family Member 2
GTF3C6	General Transcription Factor IIIC, Polypeptide 6
SLC1A5	Solute Carrier Family 1 (Neutral Amino Acid Transporter), Member 5
ERI3	ERI1 Exoribonuclease Family Member 3
SAC3D1	SAC3 Domain Containing 1

## References

[1] Moradi E., Jalili-Firoozinezhad S., Solati-Hashjin M. (2020). Microfluidic organ-on-a-chip models of human liver tissue. Acta Biomater..

[2] Samant H., Amiri H.S., Zibari G.B. (2021). Addressing the worldwide hepatocellular carcinoma: Epidemiology, prevention and management. J. Gastrointest. Oncol..

[3] Luo T., Fan L., Zhu R., Sun D. (2019). Microfluidic Single-Cell Manipulation and Analysis: Methods and Applications. Micromachines.

[4] Rumgay H., Arnold M., Ferlay J., Lesi O., Cabasag C.J., Vignat J., Laversanne M., McGlynn K.A., Soerjomataram I. (2022). Global burden of primary liver cancer in 2020 and predictions to 2040. J. Hepatol..

[5] Li Q., Cao M., Lei L., Yang F., Li H., Yan X., He S., Zhang S., Teng Y., Xia C. (2022). Burden of liver cancer: From epidemiology to prevention. Chin. J. Cancer Res..

[6] Anwanwan D., Singh S.K., Singh S., Saikam V., Singh R. (2020). Challenges in liver cancer and possible treatment approaches. Biochim. Biophys. Acta (BBA)-Rev. Cancer.

[7] World Health Organization International Agency for Research on Cancer—GLOBOCAN 2020. https://gco.iarc.fr/.

[8] Lim B., Lin Y., Navin N. (2020). Advancing Cancer Research and Medicine with Single-Cell Genomics. Cancer Cell.

[9] Craig A.J., Von Felden J., Garcia-Lezana T., Sarcognato S., Villanueva A. (2020). Tumour evolution in hepatocellular carcinoma. Nat. Rev. Gastroenterol. Hepatol..

[10] Zheng H., Pomyen Y., Hernandez M.O., Li C., Livak F., Tang W., Dang H., Greten T.F., Davis J.L., Zhao Y. (2018). Single-cell analysis reveals cancer stem cell heterogeneity in hepatocellular carcinoma. Hepatology.

[11] Javanmard D., Najafi M., Babaei M.R., Niya M.H.K., Esghaei M., Panahi M., Tameshkel F.S., Tavakoli A., Jazayeri S.M., Ghaffari H. (2020). Investigation of *CTNNB1* gene mutations and expression in hepatocellular carcinoma and cirrhosis in association with hepatitis B virus infection. Infect. Agents Cancer.

[12] Kanabekova P., Kadyrova A., Kulsharova G. (2022). Microfluidic Organ-on-a-Chip Devices for Liver Disease Modeling In Vitro. Micromachines.

[13] Ding Y., Howes P.D., Demello A.J. (2020). Recent Advances in Droplet Microfluidics. Anal. Chem..

[14] Zhang Y., Song J., Zhao Z., Yang M., Chen M., Liu C., Ji J., Zhu D. (2020). Single-cell transcriptome analysis reveals tumor immune microenvironment heterogenicity and granulocytes enrichment in colorectal cancer liver metastases. Cancer Lett..

[15] Xu Y., Zhou X. (2018). Applications of single-cell sequencing for multiomics. Methods in Molecular Biology.

[16] Feng M., Pan Y., Kong R., Shu S. (2020). Therapy of Primary Liver Cancer. Innovation.

[17] Chiang J.Y.L., Ferrell J.M. (2018). Bile Acid Metabolism in Liver Pathobiology. Gene Expr..

[18] Dimitriou P., Li J., Tornillo G., McCloy T., Barrow D. (2021). Droplet Microfluidics for Tumor Drug-Related Studies and Programmable Artificial Cells. Glob. Chall..

[19] Sliwkowski M.X., Mellman I. (2013). Antibody Therapeutics in Cancer. Science.

[20] Tavakoli H., Zhou W., Ma L., Perez S., Ibarra A., Xu F., Zhan S., Li X. (2019). Recent advances in microfluidic platforms for single-cell analysis in cancer biology, diagnosis and therapy. TrAC Trends Anal. Chem..

[21] Sohn W., Lee H.W., Lee S., Lim J.H., Lee M.W., Park C.H., Yoon S.K. (2020). Obesity and the risk of primary liver cancer: A system-atic review and meta-analysis. Clin. Mol. Hepatol..

[22] Petrick J.L., McGlynn K.A. (2019). The Changing Epidemiology of Primary Liver Cancer. Curr. Epidemiol. Rep..

[23] Dhanasekaran R., Bandoh S., Roberts L.R. (2016). Molecular pathogenesis of hepatocellular carcinoma and impact of therapeutic advances. F1000Research.

[24] Salehiniya H., Mohammadian M., Mahdavifar N., Mohammadian-Hafshejani A., Salehiniya H. (2018). Liver cancer in the world: Epidemiology, incidence, mortality and risk factors. World Cancer Res. J..

[25] Litzenburger U.M., Buenrostro J.D., Wu B., Shen Y., Sheffield N.C., Kathiria A., Greenleaf W.J., Chang H.Y. (2017). Single-cell epigenomic variability reveals functional cancer heterogeneity. Genome Biol..

[26] Heinrich S., Craig A.J., Ma L., Heinrich B., Greten T.F., Wang X.W. (2021). Understanding tumour cell heterogeneity and its implication for immunotherapy in liver cancer using single-cell analysis. J. Hepatol..

[27] Zhang M., Yang H., Wan L., Wang Z., Wang H., Ge C., Liu Y., Hao Y., Zhang D., Shi G. (2020). Single-cell transcriptomic architecture and intercellular crosstalk of human intrahepatic cholangiocarcinoma. J. Hepatol..

[28] Tian B., Li Q. (2022). Single-Cell Sequencing and Its Applications in Liver Cancer. Front. Oncol..

[29] Tang X., Huang Y., Lei J., Luo H., Zhu X. (2019). The single-cell sequencing: New developments and medical applications. Cell Biosci..

[30] Ranzoni A.M., Strzelecka P.M., Cvejic A. (2019). Application of single-cell RNA sequencing methodologies in understanding haematopoiesis and immunology. Essays Biochem..

[31] Li Y., Wu J., Li E., Xiao Z., Lei J., Zhou F., Yin X., Hu D., Mao Y., Wu L. (2022). TP53 mutation detected in circulating exosomal DNA is associated with prognosis of patients with hepatocellular carcinoma. Cancer Biol. Ther..

[32] Carter B., Zhao K. (2021). The epigenetic basis of cellular heterogeneity. Nat. Rev. Genet..

[33] Martire S., Banaszynski L.A. (2020). The roles of histone variants in fine-tuning chromatin organization and function. Nat. Rev. Mol. Cell Biol..

[34] Klemm S.L., Shipony Z., Greenleaf W.J. (2019). Chromatin accessibility and the regulatory epigenome. Nat. Rev. Genet..

[35] Monti P., Menichini P., Speciale A., Cutrona G., Fais F., Taiana E., Neri A., Bomben R., Gentile M., Gattei V. (2020). Heterogeneity of TP53 Mutations and P53 Protein Residual Function in Cancer: Does It Matter?. Front. Oncol..

[36] Rocca V., Blandino G., D’antona L., Iuliano R., Di Agostino S. (2022). Li–Fraumeni Syndrome: Mutation of *TP53* Is a Biomarker of Hereditary Predisposition to Tumor: New Insights and Advances in the Treatment. Cancers.

[37] Nge P.N., Rogers C.I., Woolley A.T. (2013). Advances in Microfluidic Materials, Functions, Integration, and Applications. Chem. Rev..

[38] Matuła K., Rivello F., Huck W.T.S. (2020). Single-Cell Analysis Using Droplet Microfluidics. Adv. Biosyst..

[39] El Debs B., Utharala R., Balyasnikova I.V., Griffiths A.D., Merten C.A. (2012). Functional single-cell hybridoma screening using droplet-based microfluidics. Proc. Natl. Acad. Sci. USA.

[40] Zilionis R., Nainys J., Veres A., Savova V., Zemmour D., Klein A.M., Mazutis L. (2017). Single-cell barcoding and sequencing using droplet microfluidics. Nat. Protoc..

[41] Srisa-Art M., Bonzani I.C., Williams A., Stevens M.M., Demello A.J., Edel J.B. (2009). Identification of rare progenitor cells from human periosteal tissue using droplet microfluidics. Analyst.

[42] Tan Y.-C., Fisher J.S., Lee A.I., Cristini V., Lee A.P. (2004). Design of microfluidic channel geometries for the control of droplet volume, chemical concentration, and sorting. Lab Chip.

[43] Aliya S., Lee H., Alhammadi M., Umapathi R., Huh Y.S. (2022). An Overview on Single-Cell Technology for Hepatocellular Carcinoma Diagnosis. Int. J. Mol. Sci..

[44] Tang H., Yuan J., Gong Y.-F., Zhang C.-Y., Liu M., Luo S.-X. (2022). Single-cell transcriptome sequencing reveals potential novel combination of biomarkers for antibody-based cancer therapeutics in hepatocellular carcinoma. Front. Genet..

[45] Dong X., Wang F., Liu C., Ling J., Jia X., Shen F., Yang N., Zhu S., Zhong L., Li Q. (2021). Single-cell analysis reveals the intra-tumor heterogeneity and identifies MLXIPL as a biomarker in the cellular trajectory of hepatocellular carcinoma. Cell Death Discov..

[46] Dechassa M.L., Tryndyak V., de Conti A., Xiao W., Beland F.A., Pogribny I.P. (2018). Identification of chromatin-accessible domains in non-alcoholic steatohepatitis-derived hepatocellular carcinoma. Mol. Carcinog..

[47] Tang L., Wang M., Shen C., Wen L., Li M., Wang D., Zheng X., Sheng Y., Wu W., Zhang C. (2021). Assay for Transposase-Accessible Chromatin Using Sequencing Analysis Reveals a Widespread Increase in Chromatin Accessibility in Psoriasis. J. Investig. Dermatol..

[48] Chen H., Lareau C., Andreani T., Vinyard M.E., Garcia S.P., Clement K., Andrade-Navarro M.A., Buenrostro J.D., Pinello L. (2019). Assessment of computational methods for the analysis of single-cell ATAC-seq data. Genome Biol..

[49] Kumar P., Kiran S., Saha S., Su Z., Paulsen T., Chatrath A., Shibata Y., Shibata E., Dutta A. (2020). ATAC-seq identifies thousands of extrachromosomal circular DNA in cancer and cell lines. Sci. Adv..

[50] Zhao Y., Zhang X., Song Z., Wei D., Wang H., Chen W., Sun G., Ma W., Chen K. (2020). Bibliometric Analysis of ATAC-Seq and Its Use in Cancer Biology via Nucleic Acid Detection. Front. Med..

[51] Ma S., Zhang Y. (2020). Profiling chromatin regulatory landscape: Insights into the development of ChIP-seq and ATAC-seq. Mol. Biomed..

[52] Buenrostro J.D., Wu B., Chang H.Y., Greenleaf W.J. (2015). ATAC-seq: A method for assaying chromatin accessibility genome-wide. Curr. Protoc. Mol. Biol..

[53] Venkatesh S., Workman J.L. (2015). Histone exchange, chromatin structure and the regulation of transcription. Nat. Rev. Mol. Cell Biol..

[54] Chen C., Liu J., Chen Y., Lin A., Mou W., Zhu L., Yang T., Cheng Q., Zhang J., Luo P. (2023). Application of ATAC-seq in tumor-specific T cell exhaustion. Cancer Gene Ther..

[55] Mun S.J., Kim J.-H., Son M.J., Kim S.-Y. (2023). Integrative analysis of single-cell RNA-seq and ATAC-seq reveals heterogeneity of induced pluripotent stem cell-derived hepatic organoids. iScience.

[56] Craig A.J., Silveira M.A.D., Ma L., Revsine M., Wang L., Heinrich S., Rae Z., Ruchinskas A., Dadkhah K., Do W. (2023). Genome-wide profiling of transcription factor activity in primary liver cancer using single-cell ATAC sequencing. Cell Rep..

[57] Baek S., Lee I. (2020). Single-cell ATAC sequencing analysis: From data preprocessing to hypothesis generation. Comput. Struct. Biotechnol. J..

[58] Xu H., Yu H., Zheng F., Zhang C., Cai W., Zhang X., Tang D., Dai Y. (2022). Analyzing the gene regulatory network in hepatitis B patients by single-cell ATAC sequencing. Clin. Rheumatol..

[59] Sun Y., Miao N., Sun T. (2019). Detect accessible chromatin using ATAC-sequencing, from principle to applications. Hereditas.

[60] Cai L.Y., Chen S.J., Xiao S.H., Sun Q.J., Ding C.H., Zheng B.N., Zhu X.Y., Liu S.Q., Yang F., Yang Y.X. (2021). Targeting p300/CBP attenuates hepatocellular carcinoma progression through epigenetic regulation of metabolism. Cancer Res..

[61] Yang H., Li G., Qiu G. (2021). Bioinformatics Analysis Using ATAC-seq and RNA-seq for the Identification of 15 Gene Signatures Associated with the Prediction of Prognosis in Hepatocellular Carcinoma. Front. Oncol..

[62] Bruix J., Gores G.J., Mazzaferro V. (2014). Hepatocellular carcinoma: Clinical frontiers and perspectives. Gut.

[63] Wang A.W., Wang Y.J., Zahm A.M., Morgan A.R., Wangensteen K.J., Kaestner K.H. (2020). The Dynamic Chromatin Architecture of the Regenerating Liver. Cell. Mol. Gastroenterol. Hepatol..

[64] Ji Z., Zhou W., Hou W., Ji H. (2020). Single-cell ATAC-seq signal extraction and enhancement with SCATE. Genome Biol..

[65] Hlady R.A., Sathyanarayan A., Thompson J.J., Zhou D., Wu Q., Pham K., Lee J., Liu C., Robertson K.D. (2019). Integrating the Epigenome to Identify Drivers of Hepatocellular Carcinoma. Hepatology.

[66] Gao D., Jin F., Zhou M., Jiang Y. (2019). Recent advances in single cell manipulation and biochemical analysis on microfluidics. Analyst.

[67] Wong A.H.-H., Li H., Jia Y., Mak P.-I., Martins R.P.d.S., Liu Y., Vong C.M., Wong H.C., Wong P.K., Wang H. (2017). Drug screening of cancer cell lines and human primary tumors using droplet microfluidics. Sci. Rep..

[68] Lin D.-C., Mayakonda A., Dinh H.Q., Huang P., Lin L., Liu X., Ding L.-W., Wang J., Berman B.P., Song E.-W. (2017). Genomic and epigenomic heterogeneity of hepatocellular carcinoma. Cancer Res..

[69] Choi S., Zhang Y., Xia Y. (2009). Fabrication of Microbeads with a Controllable Hollow Interior and Porous Wall Using a Capillary Fluidic Device. Adv. Funct. Mater..

[70] Zhou W.-M., Yan Y.-Y., Guo Q.-R., Ji H., Wang H., Xu T.-T., Makabel B., Pilarsky C., He G., Yu X.-Y. (2021). Microfluidics applications for high-throughput single cell sequencing. J. Nanobiotechnol..

[71] Tasic B. (2018). Single cell transcriptomics in neuroscience: Cell classification and beyond. Curr. Opin. Neurobiol..

[72] Macosko E.Z., Basu A., Satija R., Nemesh J., Shekhar K., Goldman M., Tirosh I., Bialas A.R., Kamitaki N., Martersteck E.M. (2015). Highly Parallel Genome-wide Expression Profiling of Individual Cells Using Nanoliter Droplets. Cell.

[73] Yang Y., Liu F., Liu W., Ma M., Gao J., Lu Y., Huang L., Li X., Shi Y., Wang X. (2020). Analysis of single-cell RNAseq identifies transitional states of T cells associated with hepatocellular carcinoma. Clin. Transl. Med..

[74] Wang Y., Wang J.-Y., Schnieke A., Fischer K. (2021). Advances in single-cell sequencing: Insights from organ transplantation. Mil. Med. Res..

[75] Gupta S., Ramesh K., Ahmed S., Kakkar V. (2016). Lab-on-Chip Technology: A Review on Design Trends and Future Scope in Biomedical Applications. Int. J. Bio-Sci. Bio-Technol..

[76] Shembekar N., Chaipan C., Utharala R., Merten C.A. (2016). Droplet-based microfluidics in drug discovery, transcriptomics and high-throughput molecular genetics. Lab Chip.

[77] Au A.K., Huynh W., Horowitz L.F., Folch A. (2016). Mikrofluidik aus dem 3D-Drucker. Angew. Chem..

[78] Chen T., Oh S., Gregory S., Shen X., Diehl A.M. (2020). Single-cell omics analysis reveals functional diversification of hepatocytes during liver regeneration. J. Clin. Investig..

[79] Balogh J., Victor D., Asham E.H., Burroughs S.G., Boktour M., Saharia A., Li X., Ghobrial R.M., Monsour H.P. (2016). Hepatocellular carcinoma: A review. J. Hepatocell. Carcinoma.

[80] Ren X., Kang B., Zhang Z. (2018). Understanding tumor ecosystems by single-cell sequencing: Promises and limitations. Genome Biol..

[81] Sima C., Hua J., Bittner M.L., Kim S., Dougherty E.R. (2018). Phenotype Classification Using Moment Features of Single-Cell Data. Cancer Inform..

[82] Burrell R.A., McGranahan N., Bartek J., Swanton C. (2013). The causes and consequences of genetic heterogeneity in cancer evolution. Nature.

